# Development and regulation of stem cell‐based therapies in China

**DOI:** 10.1111/cpr.13217

**Published:** 2022-04-13

**Authors:** Jianchao Gao, Chenyan Gao

**Affiliations:** ^1^ Office of Clinical Evaluation of Biological Products Center for Drug Evaluation, National Medical Products Administration Beijing China

## Abstract

**Background:**

Clinical researches of stem cell‐based therapies are highly active in China, while it was arduous to determine the most effective way of clinical translation of those advanced therapies.

**Methods:**

This article briefly introduced the regulatory framework development, the progress in stem cell clinical researches and clinical trials of commercially developed stem cell‐based products, as well as the clinical review concerns of stem cell‐based products in China.

**Main findings:**

The current regulatory framework of stem cell clinical researches in China was launched in 2015, when regulatory authorities issued “Administrative Measures on Stem Cell Clinical Research” (AMSCCR) detailing the rules of stem cell clinical research. Thereafter, the rapidly growing stem cell clinical researches were rigorously managed and clinical use of stem cell therapy was halted. Meanwhile, commercially developed stem cell‐based products are supervised by Drug Administration Law (DAL).

**Conclusion:**

The regulatory framework of stem cell‐based therapy in China has progressed in the last few decades, which is currently regulated according to AMSCCR and DAL. Well‐designed and patient‐focused clinical trial is required for commercially developed stem cell‐based products, and definite clinical benefit evidence is crucial to obtain marketing authorization.

## Graphical abstract

This article briefly introduced the regulatory framework development of stem cell‐based therapy, progress in stem cell‐based clinical studies and clinical review concerns in China.

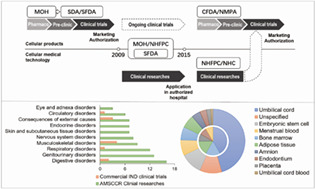

## 1 INTRODUCTION

Stem cell‐based therapies have been actively investigated from bench to clinic for decades in China,^1^ and clinical trials of stem cell‐based therapies is burgeoning in China in recent years,^2^ and therefore huge enthusiasm and expectation have been invoked by their therapeutic potentiality in a wide range of diseases and injuries with high unmet medical need. However, like in other countries and regions,^3^, ^4^, ^5^ there was substantial debate in determining the appropriate pathway for the translation and commercialization of such advanced therapeutic products in China due to the distinctions between stem cell therapies and other therapeutics like small‐molecule drugs and biologics in source origin, in vivo action, clinical regimen and healthcare readiness, etc.^6^, ^7^ In 2015, the clinical translation approach for stem cell‐based therapies was elucidated by the former Chinese National Health and Family Planning Commission (NHFPC, formerly known as Ministry of Health [MOH]; and the present National Health Commission [NHC]) and China Food and Drug Administration (CFDA, formerly known as State Drug Administration [SDA]; and the present National Medical Products Administration [NMPA]),^7^ and several technical guidelines for stem cell‐based products were drafted in the following years to meet the growing demands of biotech stakeholders and facilitate the development of stem cell‐based therapies. Herein, we briefly introduced the development and regulation framework, as well as the clinical review concerns of stem cell‐based therapies in China.

## 2 THE REGULATORY FRAMEWORK DEVELOPMENT OF STEM CELL THERAPY IN CHINA

The regulatory perception for stem cell therapy has been evolved in China for nearly three decades accompanied with developing domestic research and development (R&D) profile and deliberating on determining whether such kind of therapies should be administered as medical products.

Cell and gene therapy initially entered into the vision field of regulators in May 1993, after the implementation of ‘Primary Considerations of Quality Control in Clinical Research of Human Somatic Cell Therapy and Gene Therapy’ issued by the MOH,^8^ which interpreted that cell and gene therapy were within the regulatory boundary of Drug Administration Law (DAL), and clinical trials of such products could only be performed after being reviewed and approved by MOH. Thereafter, the application and approval procedures, as well as technical considerations were further detailed in ‘Measures for the Review and Approval of New Biological Products’ (1999)^9^ and ‘Guidelines for Research and Quality Control of Human Cell Therapeutic Products’ (2003)^10^ following the set‐up of the SDA in 1998, which was responsible for the regulation of medical products including cellular therapeutics. Since then, several cell‐based products, including mesenchymal stem cells (MSCs) and autologous immune cell, were allowed to be investigated in clinical trials. Afterwards few of those products successfully provided convincing scientific evidence of their safety and clinical benefit, and none of them has ever been approved for marketing. Meanwhile, many medical institutions and physicians circumvented the regulations by covertly treating patients with unproved stem cell therapies and falsely claiming their therapies research, causing patient confusion and driving out rigorously monitored clinical trials necessary for evaluating the benefit‐risk profile of stem cell products.^6^

The regulatory framework on stem cell therapy was acclimatized to medical technology after the implementation of ‘Regulations on the Clinical Application of Medical Technologies’ by MOH in March 2009, which specified that all kinds of stem cell‐based therapies were categorized as ‘class III’ medical technologies—those deemed ‘ethically problematic and still in need of clinical verification’.^6^ According to this regulation, any ‘class III’ medical technologies should be verified by clinical trials and go through the review process of MOH prior to clinical use, and medical institutions could not apply any ‘class III’ medical technologies only if they have been examined and approved by MOH.^11^ Meanwhile, MOH released the list of ‘class III’ medical technologies permitted for clinical application in June 2009, which included cell therapies like autoimmune cell (T cell, NK cell) therapy and haematopoietic stem cell transplantation.^12^ Although not included in the permission list and should be halted according to the regulation, stem cell‐based therapies other than haematopoietic stem cells continued to grow in the following period.^13^ In order to regularize the disrupted situation, the MOH proposed nationwide moratorium on stem cell clinical studies and therapies, and call for a one‐year special action focus on inspecting and rectifying the unproved clinical studies or applications of stem cell therapies.^14^

The new era of regulation on stem cell therapy started in July 2015, when CFDA and NHFPC jointly issued ‘Administrative Measures on Stem Cell Clinical Research’ (AMSCCR) detailing the rules of stem cell clinical research. The measures, as the first official document focusing on the administration of stem cell clinical research in China, abolished the certification and clinical use of stem cell‐based ‘class III’ medical technologies, and specified that hospital‐prepared stem cell clinical research could only be performed in competent class 3A medical institutions (the top‐level hospitals in China) which have drug clinical trial qualification as well as legal and sufficient funding support for stem cell research. Medical institutions, which are responsible for stem cell preparations and clinical research, should establish quality control system for stem cells, and organize competent academic and ethics committees suitable for reviewing and supervising stem cell clinical research projects.^15^ The institutions must neither charge subjects for participating in clinical research, nor publish or disguise stem cell clinical research advertisements.

**FIGURE 1:**
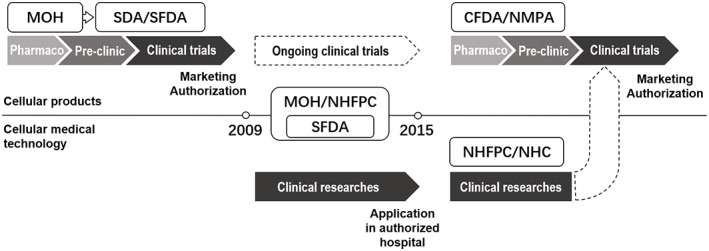
The regulatory framework development of cellular medicine in China. There were several government reconstructions in recent decades. SFDA was affiliated to MOH from 2008 to 2013. CFDA, China Food and Drug Administration; MOH, Ministry of Health; NHC, National Health Commission; NHFPC, National Health and Family Planning Commission; NMPA, National Medical Products Administration; SDA, State Drug Administration; SFDA, State Food and Drug Administration

According to AMSCCR, investigators initiating stem cell clinical researches should file materials about the medical institutions and clinical research projects to NHFPC and CFDA, and register the clinical researches online at the Chinese Medicine Registry and Management System.^7^ The NHFPC and CFDAs jointly established national expert and ethics committees on stem cell clinical research to review the materials, and conduct on‐site supervision in coordination with provincial branches of such committees. After the file materials have been accepted by the committees, the clinical research can go ahead. Notably, clinical usage of hospital‐prepared stem cell therapy after the clinical research is not allowed. The clinical study results can be submitted as research evidence to CFDA and used for evaluation in commercial investigational new drug (IND) application requests. Besides, the AMSCCR were not applicable to haematopoietic stem cell transplantation with experienced technical specifications and the clinical trials of stem cells products declared directly to the CFDA.^16^ The commercial INDs and clinical trials of stem cell‐based products are supervised by DAL and attached CFDA‐released implementation measures of DAL (Figure 1).

## 3 OVERVIEW OF THE STEM CELL CLINICAL RESEARCHES REGISTERED IN MHC AND CLINICAL TRIALS OF STEM CELL‐BASED PRODUCTS APPROVED BY NMPA

**FIGURE 2:**
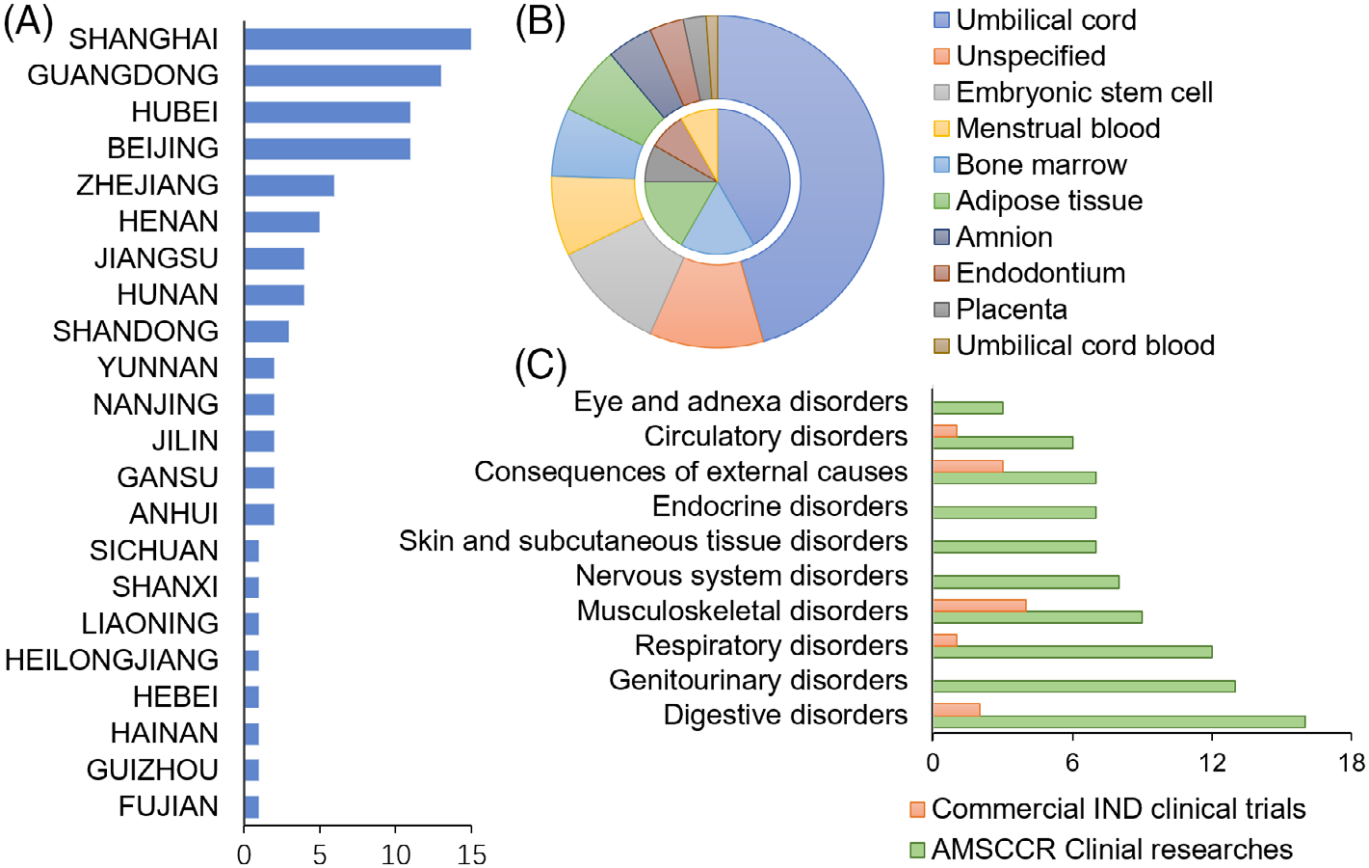
Overview of AMSCCR clinical researches and clinical trials of stem cell‐based products by the end of 2020. (A) Geographical distribution of AMSCCR clinical researches. (B) The origins of stem cells in AMSCCR clinical researches (outer ring) and commercial IND clinical trials (inner circle). (C) The patient populations explored in stem cell researches. AMSCCR, Administrative Measures on Stem Cell Clinical Research; IND, investigational new drug

By the end of 2020, more than 100 medical institutions and stem cell clinical research projects have successfully registered according to AMSCCR. These clinical researches are carried out in more than half of the provinces in China, and most abundant in east China including Shanghai, Guangdong, Beijing and Hubei, etc. (Figure 2A) MSCs are explored in vast majority (83.3%) of clinical researches, other types of cells include neuronal stem cells, retinal pigment epithelial cells, amniotic epithelial cells, bronchial basal cells and megakaryocyte progenitor cells. The MSCs mostly derived from umbilical cord tissue and placenta tissue, bone marrow, menstrual blood and adipose tissue, and there are also other sources including embryonic stem cells and dental pulp (Figure 2B). Whereas the derivation of other stem cells except MSCs included embryonic stem cells, amniotic tissue and autologous tissue like bronchial basal layer. There are a wide variety of indications investigated in those stem cell‐based clinical studies, which generally involve diseases of various organ systems, mainly of digestive system, genitourinary system, respiratory system, nervous system, skin and subcutaneous tissue, endocrine system, circulatory system, musculoskeletal system and connective tissue, etc. Since the outbreak of COVID‐19 pandemic, stem cell‐based therapies have also been tested in the treatment of severe COVID‐19 related pneumonia and acute respiratory distress syndrome. While notably, in contrast to tumour‐focusing tendency of immune cell therapies, malignant tumours are seldomly addressed in those stem cell‐based clinical studies (Figure 2C).

On the other way, the R&D of stem cell‐based products is burgeoning in China. NMPA has initiated regulatory science action on stem cell and gene therapy products since 2019 to improve the regulatory framework for such advanced therapeutic products, thereafter several guidance concerning the development of stem cell‐based products has been released^17^, ^18^, ^19^ or planning. Since the implementation of AMSCCR, dozens of stem cell‐based product sponsors have communicated with Center for Drug Evaluation (CDE) of NMPA, which is in charge of the review and evaluation of investigational medicines. The clinical trial applications of more than 10 MSC‐based product‐derived from umbilical cord, bone marrow, adipose, placenta, menses or dental pulp‐have been authorized by CDE. Notably, most of these projects have also registered according to AMSCCR, and the spectrum of indications explored in these clinical trials‐including knee osteoarthritis, graft versus host disease, diabetic foot ulcer, ischemic stroke, ulcerative colitis, chronic periodontitis or idiopathic pulmonary fibrosis‐is congruous to their counterpart clinical studies.

Although the information originated from these registered stem cell‐based clinical studies is not indubitably accepted as evidence for pharmaceutical evaluation as specified by AMSCCR, CDE has generally acknowledged the value of such information in preliminarily understanding the clinical benefit‐risk profile of tested products and developing marketing‐oriented clinical trial program.^20^ Dose‐finding trial, which is requisite for ordinary IND application, might not be necessarily rerun if the dose‐escalation effect of certain stem cell‐based products has discovered in clinical studies registered according to AMSCCR.^19^ Actually, there are few stem cell‐based investigational products were allowed to proceed into phase II clinical trial based on their safety information acquired in AMSCCR researches.

## 4 CONSIDERATIONS FOR CLINICAL TRIALS OF STEM CELL‐BASED PRODUCTS IN CHINA

Definite clinical benefit is the prerequisite for marketing authorization of stem cell‐based products in China. Cellular and gene therapy products and other drug products generally share common rationales and purposes in different phases of clinical trials, that is, to explore the dose escalation, safety and tolerance of investigational products in early phase clinical trials, afterwards regimen and benefit‐risk profile in intended populations in late confirmatory clinical trials.^21^ However, there are also notably distinct considerations for the clinical trials of stem cell‐based products.

### 4.1 Ethical review

The ethical consensus on human biomedical research have been established,^22^ while as compared to other drug products, there are more ethical dilemmas in human stem cell research regarding donation of biological materials, cell origins, and oversight of stem cell clinical trials, etc.^23^ Academic societies, such as the International Society for Stem Cell Research, offer evolving scientific perspectives on the fundamental ethical principles of stem cell research, for example, research enterprise integrity, voluntary informed consent, patient welfare, transparency and social Justice,^24^ which were also addressed in the ethical review of stem cell clinical researches in China, as specified in guidance for the construction of ethical review committee for clinical research involving humans.^25^

### 4.2 First‐in‐human study

Pre‐clinical proof‐of‐concept study is critical to understand the biological characteristics of stem cell‐based products, such as action mechanism, proliferation, differentiation and migration, as well as safety concerns like tumorigenicity, and is of great significance to determine the delivery scheme of clinical research and predict the distribution and action in human body, although cross‐species physiological difference and immunological incompatibility might be obstacles to accurately infer the cell behaviour in vivo. The creation of a more informative set of animal models for stem cell‐based therapies might contribute to overcome such shortcomings.^26^

First‐in‐human clinical trial should begin cautiously with dose‐escalation protocols and interval administration to avoid simultaneous exposure of subjects to unreasonable complications. For stem cell‐based products which should be delivered to the lesion area instead of systemic administration, the feasibility and biocompatibility of delivery system, for example, intravascular catheterization or surgical injection for heart and central nervous system disorders, should also be evaluated jointly with the cells. Furthermore, clinical tracking of living cells is proposed as it is conducive to exhibit the biodistribution, transplantation and clearance of stem cells in vivo.^27^

### 4.3 Benefit‐risk evaluation

Stem cell‐based products have been investigated in a wide range of indications in China. Endpoints consistent with clinical practice guidelines or consensus are recommended in clinical benefit evaluation. Unverified or alternative endpoints, for example, changes in cytokines or biomarkers, reduction or cessation of concomitant treatments, are generally not accepted as the primary efficacy evaluation criteria in confirmatory clinical trials, unless the correlation between such endpoints with patients' clinical benefits has been confirmed.

As a living medicine, stem cell‐based products usually survive for extended time periods in vivo; and repeated administration of allogeneic stem cell‐based products might induce immune response, thereby reducing their effectiveness and/or increasing safety risk. Therefore, the duration of efficacy is concerned in benefit‐risk evaluation of stem cell‐based products, and continuous monitoring and prolonged follow‐up are necessary to avoid the possibility of delayed reactions, for example, tumour formation after treatments.^28^

## 5 CONCLUSION

In recent years, stem cell‐based therapy has become a very promising biomedical research topic in China. The regulatory framework in China has been evolving in the past few decades, along with a deepening understanding of the scientific, social and ethical aspects of stem cell research. Although what is the most effective way to supervise and promote the development of stem cell therapy is still far from perfect in China and many other countries, patient‐focused assessment is critical for patient protection and clinical benefit‐risk evaluation in a well‐designed regulatory system.

## AUTHOR CONTRIBUTIONS

Both authors conceived the presented idea; G.J.C collected the data and wrote the paper; G.C.Y designed the analysis and modified the manuscript.

## Data Availability

The authors confirm that the data supporting the findings of this study are available within the article.
